# Health-related quality of life in migrant preschool children

**DOI:** 10.1186/1471-2458-13-384

**Published:** 2013-04-25

**Authors:** Jardena Puder, Ana Margarida Pinto, Antoine Bonvin, Patrick Bodenman, Simone Munsch, Susi Kriemler, Pedro Marques-Vidal

**Affiliations:** 1 Service of Endocrinology, Diabetes and Metabolism, Centre Hospitalier Universitaire Vaudois, University of Lausanne, Lausanne, Switzerland; 2 Institute of Preventive Medicine; Medical Faculty of Lisbon, University of Lisbon, Avenida Professor Egas Moniz, Lisbon, 1649-028, Portugal; 3 Institute of Sport Sciences, University of Lausanne, Lausanne, Switzerland; 4 Policlinique Médicale Universitaire, Unité des populations Vulnérables (UPV), Rue du Bugnon 44, Lausanne, CH-1011, Switzerland; 5 Department of Psychology, University of Fribourg, Fribourg, Switzerland; 6 Swiss Tropical and Public Health Institute (STPH), University of Basel, Basel, Switzerland; 7 Institute of Social and Preventive Medicine (IUMSP), University of Lausanne, Route de la Corniche 10, Lausanne, Switzerland; 8 Institut Universitaire de Médecine Sociale et Préventive, Bâtiment Biopôle 2, Route de la Corniche 10, Lausanne, CH-1010, Switzerland

**Keywords:** Quality of life, Children, Migrants, Switzerland, PedsQL, HRQOL, Ballabeina, Youp’là Bouge

## Abstract

**Background:**

Minority groups have a lower health-related quality of life (HRQOL), but there is little information if this finding also applies to children. In this study, we compared HRQOL between young children with and without migrant parents.

**Methods:**

Two cross-sectional studies of culturally diverse preschool populations in Switzerland: Ballabeina (40 preschools, 258 girls and 232 boys aged 4 to 6 years) and Youp’là Bouge (58 child care centers, 453 girls and 522 boys aged 2 to 4 years). Most children were born in Switzerland (Ballabeina: 92.3%; Youp’là Bouge: 93.7%). Number of migrant parents was considered as the main exposure. HRQOL was measured using the 23-item Pediatric Quality of Life Inventory.

**Results:**

Children of migrant parents had a significantly lower HRQOL total score (mean ± SD, Ballabeina: 84.2 ± 9.1; 82.7 ± 9.6 and 81.7 ± 11.7 for children with none, one or two migrant parents, respectively; Youp’là Bouge: 83.8 ± 8.6; 82.9 ± 9.5; 80.7 ± 11.7, all p < 0.05). Similar results were found in Ballabeina and Youp’là Bouge for social, school and physical functioning (all p < 0.05), but not for emotional functioning. The differences in HRQOL measures were partly mediated by children’s place of birth, parental education, paternal occupational level, children’s BMI, screen time and physical activity in one study (Ballabeina), but not in the other (Youp’là Bouge).

**Conclusion:**

In preschoolers, children of migrant parents have lower HRQOL than children of non-migrant parents. These differences are only partly mediated by other sociocultural characteristics or lifestyle behavior. These families may need assistance to prevent further inequalities.

## Background

Health-related quality of life (HRQOL) is a multidimensional construct that includes physical, emotional and social health dimensions as delineated by the WHO [1]. In adults, HRQOL has been shown to predict mortality [2,3]. In children, HRQOL is an important indicator of everyday functioning and any relevant reductions in these functions are critical to the child’s well-being [4].

In the US, minority groups have been found to have a lower quality of life [5,6], but there is little data extending these findings to schoolchildren and adolescents [7,8]. In view of the increasing proportion of the migrant populations in Europe and worldwide [9] and the known health disparities as a function of migrant status [10], it is thus crucial to measure HRQOL in migrants. Several studies have demonstrated a decrease in the HRQOL in specific adult migrant populations compared to the native population [11,12]. Conversely, and to the best of our knowledge, only one study conducted in Spain addressed HRQOL in migrant adolescents. In this study, migrant adolescents had a worse HRQOL than adolescents born in Spain [13]. Conversely, a study conducted in the USA found no difference between itinerant and geographically stable children [14]. It is also possible that parental migrant status might influence children’s well-being. This may be mediated through differences in lifestyle behaviors of the children, the socioeconomic situation of the family, access to health care or cultural and language gaps [15]. However, we are not aware of any study investigating the impact of parental migration on HRQOL in children.

Thus, we aimed to compare HRQOL between young children with and without migrant parents using data from two separate culturally diverse populations of 2- to 4- and 4-to 6-year-old preschoolers.

## Methods

### Study design

Baseline data from the Ballabeina and the Youp’là Bouge studies were used. Both studies were approved by the respective cantonal ethical committees (Vaud, St. Gallen and Basel for Ballabeina and Vaud, Neuchâtel and Jura for Youp’là bouge) and written informed consent was provided from the parents or legal representatives of each child. No child was excluded from study participation.

The Ballabeina study (clinicatrials.gov NCT00674544) is a multicenter cluster-randomized lifestyle intervention trial performed between 2008 and 2009 [16]. The study randomly selected 40 classes from the French and German part of Switzerland, focusing on areas with a high migrant prevalence (≥40%).

**Figure 1:**
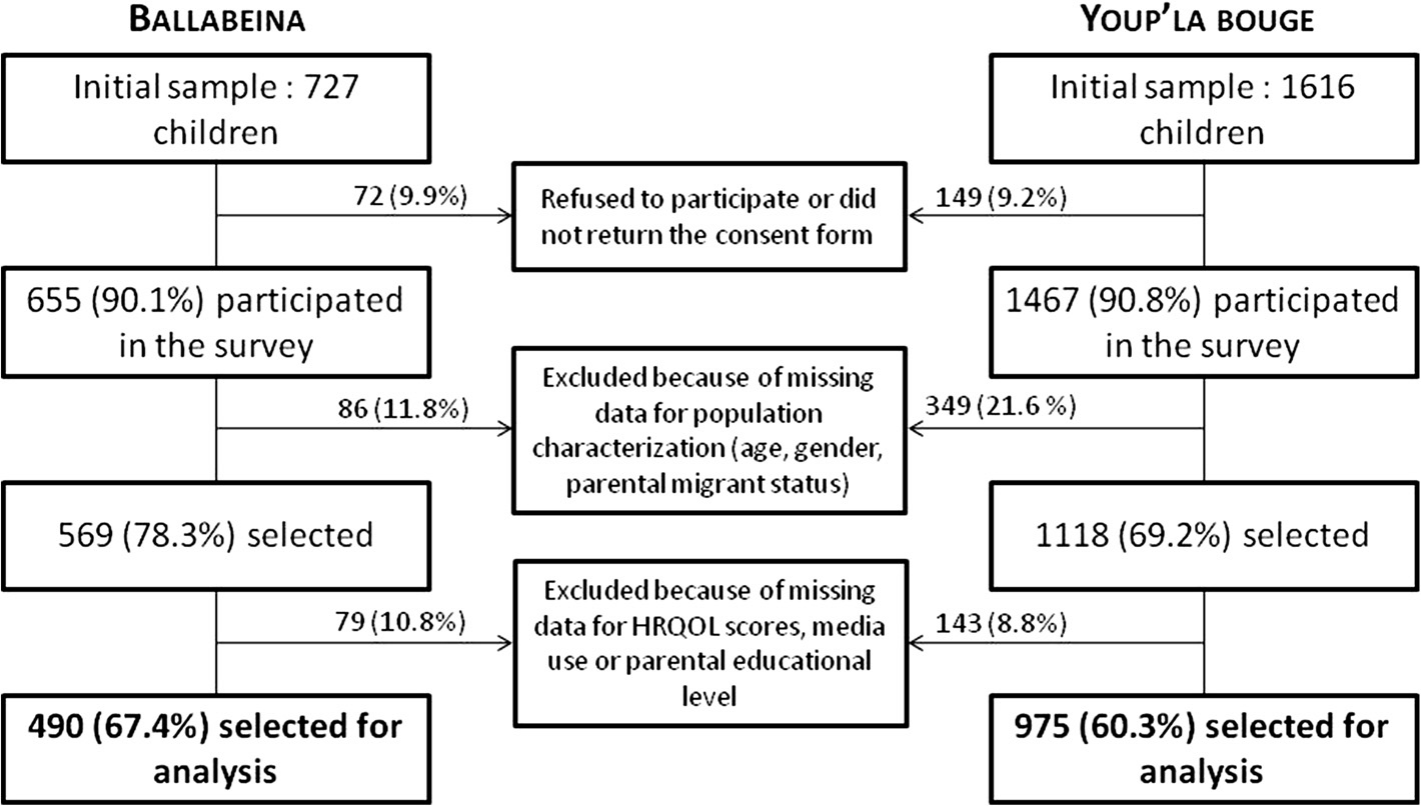
**Sample selection for analysis.** Percentages were calculated using initial sample as denominator.

The Youp’là Bouge study (clinicatrials.gov NCT00967460) is also a multicenter cluster-randomized physical activity intervention trial in childcare centers performed between 2009 and 2010 [17]. The study randomly selected 58 child care centers from the French part of Switzerland. The flow chart summarizing participant selection for both studies is provided in Figure 1. Only children with data for age, gender, parental migrant status, educational level and HRQOL were selected for the present analysis.

### Measures

Data on sociocultural characteristics, lifestyle behaviors and HRQOL were collected by questionnaire. Questionnaires were available in German or French. In the case parents had difficulties in understanding German or French, teachers and translators were available for assistance. Only 5 parents asked for assistance.

### Health-related quality of life

In both studies, the HRQOL of the children was assessed by the parent proxy version of the PedsQL™ 4.0 (Pediatric Quality of Life Inventory) Generic Core Scales questionnaire. This questionnaire consists of 23 items and contains four scales that assess the physical, emotional, social and school dimensions [16,18,19]. Respondents are asked how much of a problem each item has been during the past 1 month. A Likert scale of 5 response options (never, almost never, sometimes, often, and almost always a problem) was used, with higher scores indicating better HRQOL [16,18,19]. The PedsQL™ 4.0 has been demonstrated to be a feasible and reliable, well-validated instrument both in healthy and patient populations [7,18-20] as well as in school settings [7]. It has been studied in different European countries and in the US and has cross-cultural applicability [18,21,22].

The Physical Health Summary Score of the PedsQL™ 4.0 is the same as the physical functioning scale. Examples for physical functioning are walking more than a block, running, lifting something heavy or low energy level. The Psychosocial Health Summary Score corresponds to the mean of the Emotional, Social and School Functioning scales. Examples for emotional functioning are feeling afraid or scared or feeling angry; examples for social functioning are getting along with other children or keeping up when playing with other children; examples for school functioning are paying attention in class, forgetting things or missing school because of not feeling well.

In the Ballabeina study, the parent proxy-report for ages 5–7 of the PedsQL^TM^ 4.0 was used. In the Youp’là bouge study, the parent proxy-report for ages 2–4 was used. Depending on the region, the validated German and/or French translations were used. Given the limited resources, the child self-report for ages 5–7 was not collected as it requires individual interviewer administration [7].

### Sociocultural characteristics

Parental migrant status was defined as being born outside of Switzerland [13,23]. Information on the length of stay in Switzerland of the migrant parents, the children’s country of birth and the language spoken at home (Ballabeina only) were also collected. Parental educational level was used as a proxy of socioeconomic status [24] and was assessed as the respective highest grade of school completed. Low educational level was defined as having completed only mandatory school (9 years). Maternal and paternal workloads were divided into two categories: less than 80% and ≥80%. Due to preschool legislation, no information could be obtained regarding income data. Three migration categories were created based on the number of migrant parents (none, one or both). The same procedure was applied to the parental educational level (number of parents with low education).

### Body mass index and lifestyle behavior

In both studies, standing height was determined and body weight was measured using an electronic scale (Seca®, Basel, Switzerland; accuracy 0.05 kg). Body mass index (BMI) was calculated as weight (kg)/height^2^ (m^2^). BMI data were only available in 488/975 children of the Youp’là Bouge study, as attendance of the children at their daycare center was 48 ± 26%. Parents were asked about their children’s daily sleep and screen time (television + computer + other electronic) and the time spent playing outside. This last item was considered as a measure of physical activity [25]. In the Ballabeina study, parents were also asked about any chronic health conditions of their children.

### Statistical methods

Statistical analysis was conducted using SPSS version 18 (SPSS Inc., Chicago, IL, USA) and Stata version 11 (Stata Corp, College Station, TX, USA). The same analytic methodology was performed for both studies. Results were expressed as number of subjects (percentage) or as mean ± standard deviation, unless otherwise stated. Bivariate analyses were conducted using chi-square or analysis of variance (ANOVA). Baseline characteristics according to the parental migrant status (i.e. none, one or two migrant parents) were compared using mixed linear or logistic regression models using the preschool class or child care center as cluster and modelling the effect as random. The association between parental migrant status and children’s HRQOL was assessed using mixed linear regression models with the respective dimensions of the PedsQL™ 4.0 scores as outcome variables, age and gender as fixed factors and preschool class or child care as cluster with random effect. Regression models were subsequently adjusted for potential confounders such as parental educational or occupational level, children’s country of birth, BMI, sleep, screen time and physical activity. In the case the association between the PedsQL™ 4.0 score and parental migrant status was modified by the confounder (for example a significant association with parental migrant status that becomes non-significant after adjusting for the potential confounder), the confounder was considered as mediating the association. The association of parental migrant status with HRQOL was also tested in the subgroup of children who did not speak a foreign (non-Swiss) language at home or who had no parents with low educational level. In the subgroup of children with at least one migrant parent, the correlation between HRQOL and parental length of stay in Switzerland was also assessed. Statistical significance was assessed for p < 0.05.

## Results

### Sampling

Of the initial 727 children from the Ballabeina study, 490 (67.4%, 258 girls, mean age 5.2 ± 0.6 years) had a complete valid dataset. Of the initial 1616 children from the Youp’là Bouge study, 975 (60.3%, 453 girls, mean age 3.3 ± 0.7 years) had a complete valid dataset (see flowchart in Figure 1).

### Participants’ characteristics

The children’s characteristics are summarized in Table 1. In both studies, sociocultural characteristics and lifestyle behavior differed according to parental migrant status.

**Table 1:** Characteristics of participants, according to the number of migrant parents, Ballabeina and Youp’là Bouge studies

	**Number of migrant parents **^**1**^			**Test (*p *value) **^**2**^	**Test (*p *value) **^**3**^
	**None**	**One**	**Both**		
Ballabeina (N)	142	124	224		
Girls (%)	76 (53.5)	65 (52.4)	117 (52.2)	0.97	0.80
Age (years)	5.1 ± 0.6	5.3 ± 0.6	5.2 ± 0.6	0.15	0.99
Born in Switzerland (%)	139 (98.6)	116 (94.3)	192 (86.5)	<0.001	<0.001
Foreign language spoken at home (%)	4 (2.9)	22 (17.7)	161 (71.9)	<0.001	<0.001
At least one parent with low education (%) ^4^	12 (8.4)	43 (34.7)	119 (53.2)	<0.001	<0.001
Percentage occupation father ≥ 80% (%)	131 (92.3)	115 (92.7)	183 (81.7)	0.002	0.002
Percentage occupation mother ≥80% (%)	10 (7.0)	18 (14.5)	52 (23.2)	<0.001	<0.001
BMI (kg/m^2^)	15.4 ± 1.2	15.7 ± 1.6	15.9 ± 1.7	0.03	0.006
Sleep duration (minutes/day)	666 ± 30	660 ± 30	648 ± 36	<0.001	<0.001
Screen time (minutes/day)	38 ± 36	69 ± 63	82 ± 64	<0.001	<0.001
Playing outside (minutes/day)	104 ± 60	88 ± 55	86 ± 54	0.01	0.11
Youp’là bouge (N)	482	275	218		
Girls (%)	229 (47.5)	134 (48.7)	90 (41.3)	0.21	0.19
Age (years)	3.3 ± 0.7	3.3 ± 0.7	3.3 ± 0.7	0.93	0.26
Born in Switzerland (%)	470 (97.5)	262 (95.3)	182 (83.5)	<0.001	<0.001
At least one parent with low education (%) ^4^	20 (4.1)	44 (16.0)	69 (31.6)	<0.001	<0.001
Percentage occupation father ≥ 80% (%)	453 (94.0)	240 (87.3)	190 (87.2)	0.001	<0.005
Percentage occupation mother ≥80% (%)	93 (19.3)	84 (30.6)	117 (53.7)	<0.001	<0.001
BMI (kg/m^2^)	16.0 ± 1.2	16.3 ± 1.3	16.6 ± 1.3	<0.001	<0.001
Sleep duration (minutes/day)	668 ± 41	656 ± 43	641 ± 44	<0.001	<0.001
Screen time (minutes/day)	31 ± 30	37 ± 35	52 ± 49	<0.001	<0.001
Playing outside (minutes/day)	115 ± 68	200 ± 65	76 ± 56	<0.001	<0.01

^1^ born outside of Switzerland; ^2^ using ANOVA (quantitative data) or chi-square test (qualitative data), not taking child care (cluster) into account; ^3^ Impact of having one additional migrant parent on different outcomes using linear (quantitative data) or logistic (qualitative data) analyses adjusting for preschool class as cluster with random effect; ^4^ at most 9 years of education. Results are expressed as mean ± standard deviation or as number of subjects (percentage).

In Ballabeina, the most important migrant countries of origin were Portugal (14%), Albania/Kosovo (13%), Former Yugoslavia (12%), Africa (11%) and other European countries (11%). Median and [interquartile range] of length of stay in Switzerland was 20 [12–35] years for migrant fathers and 17 [9–33] years for migrant mothers. Only 2.5% of the children were reported to present a chronic health condition.

In Youp’là Bouge, the most important migrant countries of origin were France (24%), Portugal (14%), Africa (14%) and other European countries (10%). Median and [interquartile range] of length of stay in Switzerland was 12 [7-20] for migrant fathers and and 10 [6-19] years for migrant mothers.

### Parental migrant status and children’s quality of life

The results of the different PedsQL™ 4.0 HRQOL scores according to the number of migrant parents are summarized in Table 2. In both studies, children with migrant parents had lower scores for total HRQOL as well as for the social, school and physical functioning. Lower scores among children of migrant parents were also found for psychosocial health in Youp’là Bouge. Parental migrant status was positively associated with the emotional functioning in Ballabeina but not in Youp’là Bouge.

**Table 2:** Health-related quality of life according to the number of migrant parents, Ballabeina and Youp’là Bouge studies

	**Number of migrant parents **^**1**^			
	**None**	**One**	**Both**	**Effect **^**2**^
Ballabeina (N)	**142**	**124**	**224**	
Emotional functioning	72.4 ± 14.0	71.9 ± 13.8	75.7 ± 15.6	1.75 (0.18, 3.31) *
Social functioning	89.0 ± 12.1	86.5 ± 12.7	83.9 ± 15.1	−2.53 (−3.96, -1.11)***
School functioning	86.5 ± 11.9	85.2 ± 13.6	83.6 ± 15.0	−1.54 (−3.02, -0.05) *
Physical health	87.2 ± 11.1	85.6 ± 13.4	83.1 ± 15.0	−2.04 (−3.49, -0.59) **
Psychosocial health	82.6 ± 9.6	81.2 ± 10.1	81.0 ± 12.0	−0.77 (−1.92, 0.39) ^NS^
Total score	84.2 ± 9.1	82.7 ± 9.6	81.7 ± 11.7	−1.23 (−2.35, -0.12)*
Youp’là Bouge (N)	**482**	**275**	**218**	
Emotional functioning	69.4 ± 13.8	68.5 ± 13.3	68.5 ± 14.5	−0.46 (−1.57, 0.65) ^NS^
Social functioning	89.2 ± 11.6	89.5 ± 11.2	86.0 ± 13.9	−1.23 (−2.18, -0.28) *
School functioning	91.9 ± 10.7	89.0 ± 12.5	85.9 ± 15.5	−2.91 (−3.88, -1.94)***
Physical health	84.1 ± 11.3	83.5 ± 12.0	81.2 ± 14.8	−1.33 (−2.30, -0.36)**
Psychosocial health	83.5 ± 8.7	82.3 ± 9.5	80.2 ± 11.6	−1.57 (−2.33, -0.82)***
Total score	83.8 ± 8.6	82.9 ± 9.5	80.7 ± 11.7	−1.45 (−2.20, -0.69)***

Impact of having one or more migrant parent(s) on the children’s quality of life. Raw data are expressed as mean ± standard deviation.^1^ born outside of Switzerland; ^2^ using mixed linear regression models with age and gender as fixed factors and preschool class/child care as cluster with random effect; results are presented as β-coefficient and (95% confidence interval). ^NS^, not significant; *, p < 0.05; **, p < 0.01; ***, p < 0.001.

Almost all HRQOL dimensions showed a gradual trend between children with no, one or two migrant parents. The mean adjusted difference in the total HRQOL score between having no migrant parents and two parents migrant was obtained by multiplying the β-coefficient for total HRQOL (as in Table 2) by two, leading to values of −2.5 (p < 0.05) for Ballabeina and −2.9 (p < 0.001) for Youp’là Bouge.

### Potential confounders

The results of the multivariate analysis adjusting for potential confounders such as parental educational level or the child’s body mass index are summarized in Table 3. In Ballabeina, different sociocultural characteristics and lifestyle behaviors mediated the association between parental migrant status and children’s HRQOL (Table 3). The association with emotional functioning was mediated by the time spent playing outside. The association with school functioning was mediated by children’s country of birth, parental educational level, paternal occupation, children’s BMI and screen time. The association of migration with physical health was mediated by parental educational level and screen time. The association of migration with total HRQOL scores was mediated by parental educational level, paternal occupation, children’s BMI and screen time in the Ballabeina study, but not in the Youp’là Bouge study. Social functioning was mediated by playing outside in the Youp’là Bouge study only. Psychosocial health was not mediated by any of those confounders. Maternal occupation and sleep duration did not mediate any associations.

**Table 3:** Impact of migration on health-related quality of life after adjustment for potential confounders, Ballabeina and Youp’là Bouge studies

**After adjustment for**		**Parental characteristics**		**Children’s characteristics**			
**After adjustment for**	**No adjustment **^**§**^	**Parental educational Level**	**Paternal occupation**	**Country of birth**	**Body mass index**	**Screen time**	**Playing outside**
Ballabeina							
Emotional functioning	1.75	2.05	1.78	1.69	1.79	2.28	1.00
	(0.18, 3.31) *	(0.33, 3.78)*	(0.21, 3.35)*	(0.11, 3.27)*	(0.20, 3.38)*	(0.65, 3.91)**	(−0.67, 2.68) ^NS^
Social functioning	−2.53	−1.60	−2.21	−2.7	−2.32	−1.92	−2.85
	(−3.96, -1.11)***	(−3.16, -0.03)*	(−3.64, -0.78)**	(−4.17, -1.24)***	(−3.76, -0.88)***	(−3.41, -0.44)*	(−4.38, -1.33)***
School functioning	−1.54	−0.61	−1.37	−1.50	−1.38	−0.65	−1.62
	(−3.02, -0.05) *	(−2.21, 1.00) ^NS^	(−2.86, 0.13) ^NS^	(−3.02, 0.02) ^NS^	(−2.89, 0.12) ^NS^	(−2.16, 0.85) ^NS^	(−3.23, -0.01)*
Physical health	−2.04	−1.10	−1.97	−1.93	−1.92	−1.27	−2.18
	(−3.49, -0.59) **	(−2.67, 0.47) ^NS^	(−3.43, -0.52)**	(−3.42, -0.45)*	(−3.38, -0.47)**	(−2.75, 0.21) ^NS^	(−3.65, -0.71)**
Psychosocial health	−0.77	−0.07	−0.59	−0.81	−0.61	−0.09	−1.15
	(−1.92, 0.39) ^NS^	(−1.33, 1.20) ^NS^	(−1.74, 0.57) ^NS^	(−1.98, 0.36) ^NS^	(−1.76, 0.55) ^NS^	(−1.28, 1.09) ^NS^	(−2.37, 0.08) ^NS^
Total score	−1.23	−0.45	−1.09	−1.24	−1.09	−0.52	−1.52
	(−2.35, -0.12)*	(−1.66, 0.77) ^NS^	(−2.21, 0.02) ^NS^	(−2.37, -0.10)*	(−2.21, 0.03) ^NS^	(−1.66, 0.62) ^NS^	(−2.67, -0.36)**
Youp’là Bouge							
Emotional functioning	−0.46	−0.76	−0.46	−0.53	NA	−0.55	−0.33
	(−1.57, 0.65) ^NS^	(−1.94, 0.41) ^NS^	(−1.58, 0.66) ^NS^	(−1.67, 0.60) ^NS^		(−1.70, 0.59) ^NS^	(−1.49, 0.83) ^NS^
Social functioning	−1.23	−1.27	−1.23	−1.18	NA	−1.14	−0.88
	(−2.18, -0.28) *	(−2.28, -0.26)*	(−2.19, -0.28)*	(−2.15, -0.20)*		(−2.11, -0.16)*	(−1.88, 0.11) ^NS^
School functioning	−2.91	−2.73	−2.84	−3.02	NA	−2.56	−2.62
	(−3.88, -1.94)***	(−3.76, -1.71)***	(−3.82, -1.86)***	(−4.01, -2.02)***		(−3.55, -1.57)***	(−3.64, -1.61)***
Physical health	−1.33	−1.70	−1.42	−1.42	NA	−1.37	−1.09
	(−2.30, -0.36)**	(−2.73, -0.68)***	(−2.39, -0.45)**	(−2.41, -0.43)**		(−2.36, -0.37)**	(−2.10, -0.08)*
Psychosocial health	−1.57	−1.64	−1.55	−1.62	NA	−1.46	−1.32
	(−2.33, -0.82)***	(−2.44, -0.83)***	(−2.32, -0.79)***	(−2.39, -0.84)***		(−2.24, -0.68)***	(−2.11, -0.53)***
Total score	−1.45	−1.66	−1.48	−1.51	NA	−1.41	−1.20
	(−2.20, -0.69)***	(−2.47, -0.86)***	(−2.24, -0.73)***	(−2.29, -0.73)***		(−2.19, -0.63)***	(−1.99, -0.40)**

Impact of having one or more migrant parent(s) on the children’s quality of life. Statistical analysis by mixed linear regression models with age and gender as fixed factors and preschool class/child care as cluster with random effect, adjusting for the respective potential confounder. ^§^ same as the last column of Table 2. Results are presented as β-coefficient and (95% confidence interval). In the case the association between the PedsQL^TM^ 4.0 score and parental migrant status is modified by the confounder (for example a significant association that becomes non-significant after adjusting for the potential confounder), the confounder is considered as mediating the association. ^NS^, not significant; *, p < 0.05; **, p < 0.01; ***, p < 0.001. NA not applicable, as data were available for half of the children only (488/975 children).

In the subgroup of 303 children who did not speak a foreign language at home, parental migrant status was still associated with all HRQOL measures except for emotional functioning (Table 4). Similarly, in the subgroup of 316 children who had no parents with low educational level, parental migrant status was still associated with total HRQOL, social functioning and physical health.

**Table 4:** Impact of migration on health-related quality of life in specific subgroups of the Ballabeina study

	**No parents with low education**	**No foreign language spoken at home**
Ballabeina (N)	316	303
Emotional functioning	1.45	0.04
	(−0.42, 3.31) ^NS^	(−2.05, 2.14) ^NS^
Social functioning	−2.84	−2.91
	(−4.47, -1.20)***	(−4.65, -1.17)***
School functioning	−1.39	−2.33
	(−3.10, 0.33) ^NS^	(−4.36, -0.30)*
Physical health	−2.12	−2.9
	(−3.77, -0.47)*	(−4.77, -1.03)**
Psychosocial health	−0.92	−1.71
	(−2.25, 0.41)	(−3.20, -0.22)*
Total score	−1.36	−2.14
	(−2.64, -0.08)*	(−3.55, -0.73)**

Impact of having one or more migrant parent(s) on the children’s quality of life in children with parents without a low educational level (column 1) or in children who do not speak a foreign language at home (column 2). Statistical analysis by mixed linear regression models with age and gender as fixed factors and preschool class as cluster with random effect. Results are presented as β-coefficient and (95% confidence interval). ^NS^, not significant; *, p < 0.05; **, p < 0.01; ***, p < 0.001.

In Youp’là bouge, none of the investigated confounders mediated the association of parental migrant status with the children’s HRQOL except for physical activity (playing outside) that mediated the association with social functioning. The association between migrant status and emotional functioning was non-significant, and further adjustment for potential confounders did not change the results.

Finally, among children with at least one migrant parent, the parent’s length of stay in Switzerland showed no significant effect on the children’s HRQOL of in both studies (Additional file 1: Table S1).

## Discussion

This study shows that preschool children with migrant parents present reduced HRQOL levels compared to preschool children for which both parents are Swiss. This decrease in HRQOL in children from migrant parents was only partly mediated by other sociocultural characteristics or the children’s lifestyle behavior.

It seems surprising that parental migration had an impact on HRQOL of such young children. This may be relevant, as this young age has been shown to be a critical period for health outcomes. Interestingly, the magnitude of the unadjusted differences between children with two and children with no migrant parents was similar in Youp’là Bouge and Ballabeina studies. Such a magnitude of differences is comparable to the differences observed between healthy children and children presenting with chronic diseases such as cardiovascular disease or diabetes [26] (Additional file 1: Table S2).

In both studies, children with migrant parents had lower levels of the physical, social and school dimension. However, children with migrant parents had higher levels of emotional functioning in the Ballabeina study, while no such association was found in the Youp’là Bouge study. This discordance between emotional functioning and the other components has not been previously reported in migrant children [8,13,22], but is in agreement with a previous study that showed a decrease in all parent-reported HRQOL dimensions except for the emotional one among schoolchildren and adolescents of low socioeconomic status [7]. Even though parents and caregivers tend to underestimate emotional problems compared to behavioral problems [27], several hypotheses may explain these observations. For example, the emotional dimension of children may be affected differently from the challenges that the migrant or socially disadvantaged families face. Migrant or socially disadvantaged parents may also recognize physical, social or school functioning difficulties in their children easier than emotional difficulties due to cultural differences in identifying and naming emotional regulation [28]. Finally, it is possible that, in these very young children, the physical, social and school problems might represent precursors of emotional problems. These problems could develop later in life as a consequence of adverse conditions in their current daily living.

These reduced HRQOL scores may have potential implications for the children’s success in social and learning environments. Although we do not know of any other study investigating HRQOL in culturally diverse migrant children, other studies focusing on school-aged children from minority groups have been conducted in the US [7,8]. For instance, Mansour *et al.* investigated mostly black urban population and noted low HRQOL scores compared to the scores of other US cohorts [8]. Another study in US schoolchildren and adolescents found lower PedsQL™ 4.0 HRQOL scores in Hispanic, Black and Asian/Pacific Islander compared to Caucasian children [7]. Using a different questionnaire, a study conducted in Spain also found that migrant adolescents had lower HRQOL [13].

The reasons for the decrease in HRQOL are most likely multifactorial, as migrants can face many challenges for health and well-being [11,29]. These are a) more vulnerable socioeconomic situations (parental income, educational achievements, family wealth); b) lower school quality for their children [8,30]; c) less access to health insurance; d) more family-based and general life events [4]; e) differences in health-related lifestyle behaviors; f) racial discrimination; g) cultural and language gaps [11]; h) lack of a cohesive social support and i) parental loss of social relations and increased psychological distress [13]*.* In the study of Mansour *et al.*, grade, employment, family income, insurance and school connectedness were associated with poorer HRQOL [8]. In the Spanish study, the impact of migration was completely mediated by socioeconomic status, social support and psychological distress [13].

In this study, we tested if the observed decrease in HRQOL among children of migrant parents was mediated by sociocultural characteristics, lifestyle and BMI. This methodology has been performed previously [4,7,18,29]. As Switzerland provides universal health coverage, no adjustment for health insurance was performed. Similarly, as the migrant and non-migrant children attended the same preschools or child care centers, no adjustment for school quality was performed.

In our study, parental educational level, paternal occupation, screen time and the children’s BMI were the most relevant counfounders. However, they only partly mediated the association of migration with HRQOL in Ballabeina and did not mediate them in Youp’là bouge. Similarly, differences in HRQOL were still present in the subgroups of children with middle/high parental educational level or who spoke one of the national languages at home in Ballabeina. Thus, although the large majority of “migrant children” was born in Switzerland, the parental migratory experience (including the loss of social relations and cultural references as well as other psychological consequences) significantly impacted their HRQOL, namely when both parents were migrants. One might thus postulate an intergenerational transmission to offspring.

The strengths of this study are the inclusion of two populations-based samples of preschool children with different age groups, the use of the same validated HRQOL questionnaire and the adjustment for relevant potential confounders such as sociocultural characteristics, lifestyle behavior and BMI. The main limitation is the fact that we only obtained proxy-report for HRQOL. Still, there is a fundamental role for parent proxy-report in young children [20], where parents’ perception of health is often what leads health-seeking behavior [8]. Further, the PedsQL self-report has only been validated in children aged 5 and older [20] and needs interviewing in this young population. Another limitation is that we had incomplete information on socioeconomic status which is composed of education, income, family wealth or occupation [23]. As we were not allowed to collect data about income and wealth, parental educational level was used as a proxy for socioeconomic status [31]. Finally, for a few children, we had to rely on the assistance of translators, which might have influenced the reporting behavior of the parents. Still, only a very small number of children (5) had data collected with the assistance of translators, and it is unlikely that this small number might have modified the associations.

## Conclusion

Our results suggest that in a culturally diverse population, children of migrant parents have lower HRQOL scores than children of non-migrant parents. The impact on HRQOL of having both parents migrant is comparable to having a chronic disease. Other sociocultural characteristics (parental educational level) and lifestyle behaviors (excessive children’s screen time) only partly mediate these differences. Thus, migrant children of parents with low educational level might seem to be more at risk and should be identified. Social and healthcare workers should be able to evaluate the situation of migrant families and assist them in order to improve the children HRQOL early on. This might be in part through adaption of certain lifestyle behaviors such as excessive screen time, through support at preschool and school or through activities (e.g. regular sports activities at a community center) that promote social integration. Pediatricians might also discuss quality of life of the family and specifically the children during the regular check-up visits. Most importantly, parents as well as health care providers should be trained in recognizing correlates of emotional problems as they risk to be underestimated in general [32]. It will be interesting to follow these children to assess how these differences in HRQOL will evolve.

## Competing interests

The authors declare that they have no competing interests.

## Authors’ contributions

AMP made most of the statistical analyses and wrote most of the article. JP conceived and designed the study, helped in data analysis and wrote part of the article. SK, PB and SM conceived and designed the study, helped in data analysis and revised the article for important intellectual content. AB collected the data and revised the article for important intellectual content. PMV made part of the statistical analyses and wrote part of the article. PMV had access to all the data and analyses and is the guarantor of the study. All authors approved the final version.

## Pre-publication history

The pre-publication history for this paper can be accessed here:

http://www.biomedcentral.com/1471-2458/13/384/prepub

## Supplementary Material

Additional file 1: Table S1 Quality of life scores according to the duration of stay in Switzerland. **Table S2**: Effect on quality of life of having both versus no parent migrants compared to selected diseases. Click here for file
