# Supplementary material for: Health-related quality of life in migrant preschool children
Source: BMC Public Health. 2013 Apr 25;13:384. doi: 10.1186/1471-2458-13-384 (PMC3641990; doi:10.1186/1471-2458-13-384)
Supplement: Additional file 1: Table S1 — Quality of life scores according to the duration of stay in Switzerland. Table S2: Effect on quality of life of having both versus no parent migrants compared to selected diseases. [file 1471-2458-13-384-S1.doc]

**Supplementary tables**

**Supplementary table 1** – Quality of life scores according to the duration of stay in Switzerland.

|  | **≤ Median** | **> Median** | **Adjusted on cluster, age and gender (1)** | **Adjusted on (1) + Media Use** | **Adjusted on (1) + Educational Level** | **Adjusted on (1) + Educational Level & Media Use** |
| --- | --- | --- | --- | --- | --- | --- |
| Ballabeina, N ^§^ | **188** | **157** | p-value | p-value | p-value | p-value |
| Emotional | 75.3 ± 14.6 | 73.2 ± 15.8 | 0.21 | 0.23 | 0.22 | 0.21 |
| Social | 84.0 ± 15.4 | 85.5 ± 13.4 | 0.47 | 0.43 | 0.36 | 0.36 |
| School | 83.3 ± 15.6 | 85.1 ± 13.4 | 0.20 | 0.17 | 0.13 | 0.14 |
| Physical | 82.8 ± 15.1 | 85.0 ± 14.1 | 0.20 | 0.16 | 0.13 | 0.13 |
| Psychosocial | 80.8 ± 11.4 | 81.2 ± 11.7 | 0.80 | 0.73 | 0.68 | 0.68 |
| Total | 81.6 ± 11.2 | 82.5 ± 11.2 | 0.48 | 0.41 | 0.35 | 0.35 |
| Youp’là Bouge, N ^§§^ | **241** | **236** |  |  |  |  |
| Emotional | 67.9 ± 13.8 | 69.3 ± 14.0 | 0.27 | 0.28 | 0.29 | 0.30 |
| Social | 87.7 ± 12.4 | 88.4 ± 12.8 | 0.47 | 0.46 | 0.46 | 0.46 |
| School | 87.4 ± 14.3 | 88.2 ± 13.6 | 0.51 | 0.45 | 0.47 | 0.41 |
| Physical | 82.6 ± 12.8 | 82.8 ± 14.0 | 0.81 | 0.83 | 0.86 | 0.87 |
| Psychosocial | 81.0 ± 10.5 | 82.0 ± 10.5 | 0.29 | 0.27 | 0.29 | 0.27 |
| Total | 81.8 ± 10.0 | 82.4 ± 11.2 | 0.50 | 0.50 | 0.53 | 0.52 |

Results are expressed as mean ± standard deviation. The other columns show the p-value of the test comparing the two groups. When both parents were migrants, only the highest value was considered; hence, the median values are higher than reported for each gender. ^§^, median=17 years; ^§§^, median=14 years.

**Supplementary table 2**: effect on quality of life of having both versus no parent migrants vs. selected diseases

|  | **Emotional** | **Social** | **School** | **Physical** | **Psychosocial** | **Total** |
| --- | --- | --- | --- | --- | --- | --- |
| Ballabeina, 4-6y ^§^ | 3.3 | -5.1 | -2.9 | -4.1 | -1.6 | -2.5 |
| Youp’là Bouge, 2-4y ^§^ | -0.9 | -3.2 | -6.0 | -2.9 | -3.3 | -3.1 |
| Uzark, CVD, 2-4y ^§§^ | -4.4 | 2.0 | - | -1.1 | -1.4 | -1.2 |
| Uzark, CVD, 5-7y ^§§^ | -3.5 | 3.4 | -2.2 | 2.3 | -0.8 | 0.3 |
| Banerjee, HIV, 11-12y ^§§^ | 0.7 | 0.7 | -0.9 | -0.8 | -3.9 | -2.6 |
| Varni, diabetes, 5-18y ^§§^ | -7.0 | 0.5 | -6.9 | -1.6 | -4.5 | -3.5 |
| Varni, cardiac disease, 5-18y ^§§^ | -5.6 | -6.4 | -9.0 | -5.3 | -7.0 | -6.4 |
| Varni, end stage renal disease, 5-18y ^§§^ | -4.2 | -6.7 | -14.2 | -12.8 | -8.3 | -9.9 |
| Varni, psychiatric disorders, NR ^§§^ | -18.0 | -12.2 | -13.2 | -6.3 | -14.5 | -11.6 |
| Varni, cancer, 5-18y ^§§^ | -7.1 | -9.6 | -12.8 | -15.6 | -9.8 | -11.9 |
| Varni, rheumatology, 5-18y ^§§^ | -11.0 | -5.1 | -11.3 | -21.5 | -9.2 | -13.5 |
| Varni, cerebral palsy, 5-18y ^§§^ | -10.7 | -14.6 | -15.5 | -23.1 | -13.8 | -17.0 |
| Varni, obesity, 5-18y ^§§^ | -10.7 | -12.6 | -6.1 | -10.0 | -9.8 | -9.8 |
| Varni, gastrointestinal, 5-18y ^§§^ | -5.4 | -0.8 | -10.8 | -6.7 | -5.7 | -6.1 |

^§^, unadjusted difference (not adjusted for cluster to be comparable to other studies) between having both and no parents migrant; ^§§^, expressed as unadjusted difference between diseased and normal children. NR, not reported.
